# Multifocal Stevens-Johnson syndrome after concurrent phenytoin and cranial and thoracic radiation treatment, a case report

**DOI:** 10.1186/1748-717X-5-49

**Published:** 2010-06-04

**Authors:** Abdullah O Kandil, Tomas Dvorak, John Mignano, Julian K Wu, Jay-Jiguang Zhu

**Affiliations:** 1 Tufts University, School of Medicine, Boston MA 02111, USA; 2 Radiation Oncology, Tufts Medical Center, Tufts University, School of Medicine, Boston, MA 02111, USA; 3 Neurosurgery, Tufts Medical Center, Tufts University, School of Medicine, Boston, MA 02111, USA; 4 Neurology, Hematology and Oncology, Tufts Medical Center, Tufts University, School of Medicine, Boston, MA 02111, USA; 5 800 Washington Street, Box 245, Tufts Medical Center, Boston, MA 02111, USA

## Abstract

A 46 year old male patient with metastatic prostate cancer developed Stevens-Johnson syndrome (SJS), initially in three well-demarcated areas on his scalp, chest and back, corresponding to ports of radiation therapy while on phenytoin. The rash spread from these locations and became more generalized and associated with pain and sloughing in the mucous lining of the mouth. There is a documented association between phenytoin administration with concurrent cranial radiation therapy and development of SJS. Erythema multiforme (EM) associated with phenytoin and cranial radiation therapy (EMPACT) is the term that describes this reaction. However, this term may not cover the full spectrum of the disease since it describes EM associated with phenytoin and only cranial radiation therapy. This case report presents evidence that SJS may be induced by radiation to other parts of the body in addition to the cranium while phenytoin is administered concomitantly. With increasing evidence that phenytoin and levetiracetam are equally efficacious for seizure treatment and prophylaxis, and since there is no link identified so far of an association between levetiracetam and SJS, we believe that levetiracetam is a better option for patients who need anticonvulsant medication(s) while undergoing radiation therapy, especially cranial irradiation.

## Background

Patients with symptomatic metastases to osseous or soft tissues are frequently offered short courses of palliative external beam radiation. In the United States, fractionation schedules of 8 Gy in 1 fraction to 37.5 Gy delivered in 15 fractions is commonly used [1]. These treatments are generally well tolerated. The most common toxicity experienced by patients is a mild and reversible irritation of cutaneous and mucosal surfaces. Patients in need of palliative radiation are often treated concurrently with glucocorticoid steroids, anti-emetic, and/or anti-epileptic drugs (AED) for relief of symptoms associated with the underlying disease process.

Phenytoin is one of the most frequently used AEDs in adult patients. Ninety percent of the drug is protein bound and it is metabolized mostly by the liver [2]. It is in the category of enzyme inducing anti-epileptic drugs (EIAED) which include carbamazepine, oxcarbazepine, fosphenytoin, phenobarbital and primidone.

Stevens-Johnson syndrome (SJS) is a rare, but severe cutaneous, cell-mediated hypersensitivity reaction that is usually induced by medication or a virus [3,4]. Historically, SJS was considered to be part of a spectrum of erythema multiforme (EM), but now it is considered clinically distinct from EM, and is part of the SJS-toxic epidermal necrolysis (SJS-TEN) spectrum. It is characterized by heterogeneous cutaneous bullous eruptions and can result in sloughing of the epidermis [5]. Early in the disease process, the epidermis becomes infiltrated with CD8 T-lymphocytes and macrophages, while the dermis shows CD4 predominance cells. It is postulated that the lymphocytes release cytokines, which mediate the inflammatory reaction and apoptosis of epithelial cells. Patients often present with a prodrome of fever, malaise, and with mucous membrane involvement [5]. Patients with 10-30% epidermal detachment are considered to have transitional SJS-TEN [5]. Patients with more than 30% epidermal detachment are classified as TEN [5]. The most frequent complications include infection (24%) and gastrointestinal bleeding (18%) [6]. Several AEDs have been associated with increased risk of developing SJS which include phenytoin, carbamazepine, lamotrigine and phenobarbital [7,8]. Certain human leukocyte antigen (HLA) types are sometimes associated with increased risk of SJS, including HLA B1502 [9].

## Case presentation

A 46 year-old Caucasian man with a history of metastatic prostate cancer presented with erythema, pruritis, vesicles and skin sloughing in three well-demarcated areas on the head, chest and back. Mucous lining in the oral cavity also exhibited sloughing and was painful. He was admitted to the hospital for observation and pain management.

In December, 2006, he was diagnosed with diffuse, bony metastatic prostate cancer (Gleason score 9/10). He had received palliative radiation therapy (20 Gy in 5 fractions) to his right hip without complications. Seven months later, he was treated with palliative radiation therapy (8 Gy in 1 fraction) to his right ribs, again without complications. On August 1, 2007, he had a craniotomy for excision of a dural based mass with pathological diagnosis of metastatic prostate cancer. On September 18, 2007, a second resection for local recurrence of cranial metastatic prostate cancer was performed. Because of pial involvement of the tumor he was started on phenytoin for seizure prophylaxis on the day of surgery. He tolerated phenytoin well, and had experienced no anticonvulsant hypersensitivity syndrome or any other side effects. While on phenytoin, he received spinal radiation (T6-11 for T7-10 metastases with mild cord compression at T8-9) from October 1 to October 17, 2007 (36 Gy) and simultaneous whole brain radiation therapy from October 3 to October 19, 2007 (36 Gy). He also started dexamethasone 4 mg TID on the first day of radiation therapy for 11 days. The dexamethasone was increased to 8 mg TID for 7 days before a taper was initiated after completion of radiation therapy. He reported a pain level of 8 out of 10 on a pain scale at the start of radiation therapy. He started transdermal fentanyl patch 200 mcg per hour in addition to oral hydromorphone 4 mg every 4 hours for break-through pain. His pain subsided to a rating of 3 out of 10.

**Figure 1:**
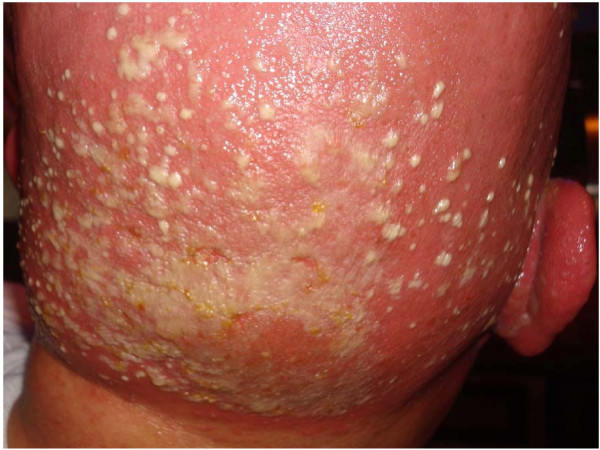
**Patient's scalp, 12 days after completion of the whole brain radiation, displaying erythematous cutaneous eruption with vesicles**.

**Figure 2:**
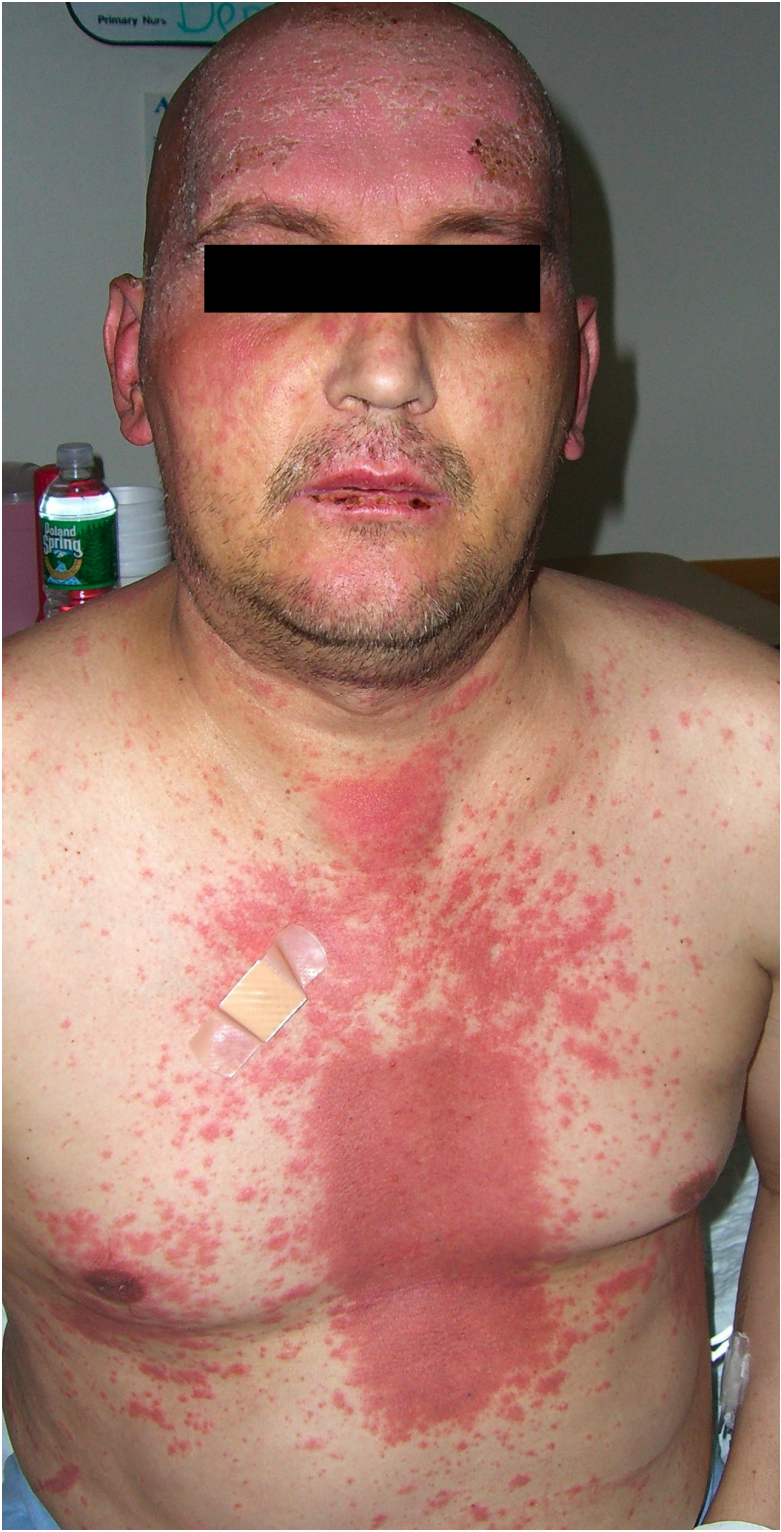
**Patient's face and chest, 14 days after completion of the spinal radiation, with erythematous cutaneous eruption, a rectangular distribution pattern, on his chest and mucous membrane involvement on his lip**.

**Figure 3:**
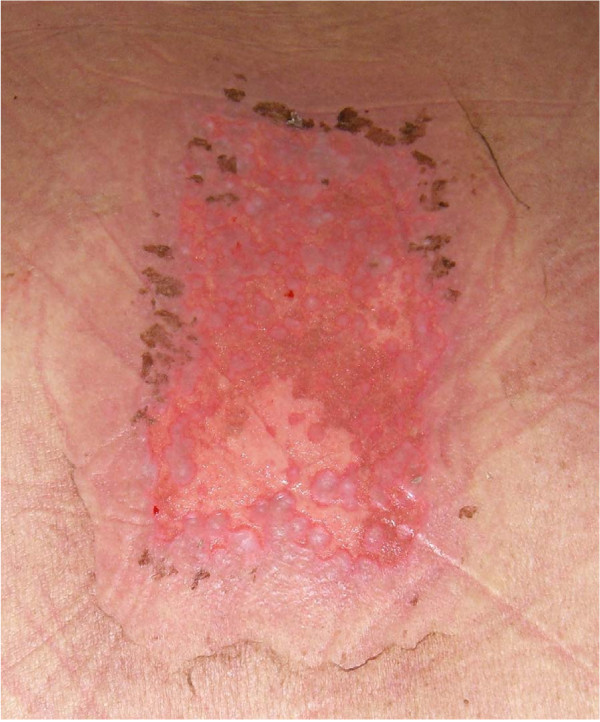
**Patient's posterior thorax, 14 days after completion of the spinal radiation therapy, showing desquamating erythematous cutaneous eruption with denuded area corresponding to the radiation port area**.

**Figure 4:**
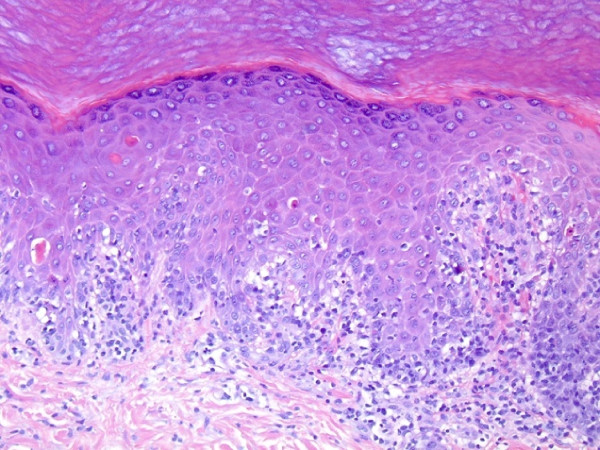
**Hematoxylin and eosin staining of palmar biopsy specimen revealing an epidermis with vacuolization of the basal layer, scattered necrotic keratinocytes and an upper dermal lichenoid mononuclear cell infiltrate**.

**Figure 5:**
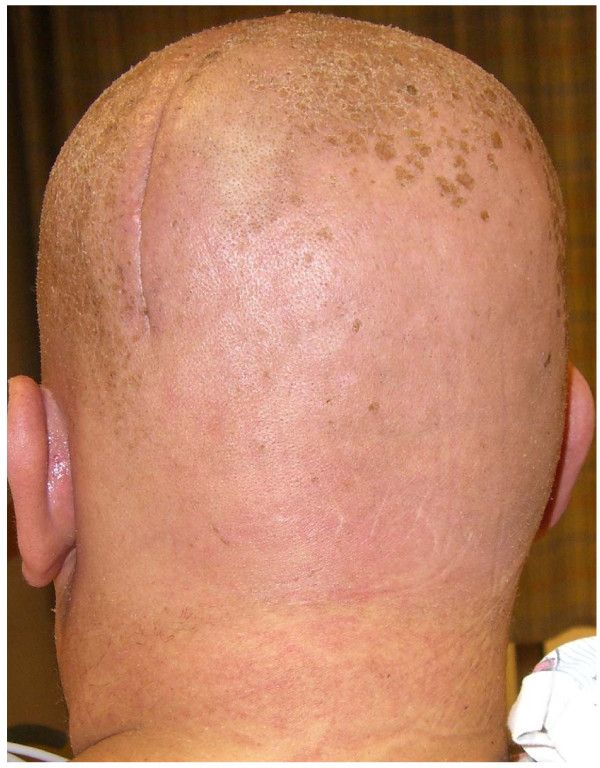
**Resolution of the SJS lesions on patient's scalp, 9 days after eruption**.

Eight days after completing radiation therapy, the patient began to develop macular and papular rashes simultaneously over the irradiated areas on his scalp and on his anterior and posterior thorax (Figures 1, 2 and 3). He was admitted to the hospital on October 29, 2007 and phenytoin was discontinued on admission. The rashes gradually became more intense, leading to vesicular pockets on the skin. The rash sloughed off in layers and the patient reported pain and pruritis. The desquamating erythematous cutaneous eruptions were originally confined only to areas of skin corresponding to the radiation port fields (Figures 1, 2 and 3). Within two days, they spread to involve most of his torso, face, as well as palmar and plantar surfaces. The most significant skin abnormalities, including enlargement of vesicles and sloughing of skin, were observed within the radiation treatment fields. The patient reported a pain level of 10 out of 10. Transdermal fentanyl patch 200 mcg per hour and oral hydromorphone 4 mg every 4 hours were resumed. He also reported fever and chills. The dexamethasone was tapered down to 1 mg twice daily. The patient was empirically treated with acyclovir and fluconazole medications. His vital signs and temperature were within normal ranges. Peripheral blood test showed elevation of prostate specific antigen (PSA) to 136.98 nanograms/ml (normal range 0.0-4.0 nanograms/ml), while other serum tests were within normal limits, including complete blood counts, electrolytes, kidney function and liver function tests. The patient was tested for HLA B1502 which was negative [9]. Palmar and chest biopsy (biopsy site on his chest was covered with bandage in Figure 2) revealed epidermis with vacuolization of the basal layer and scattered necrotic keratinocytes as well as upper dermal lichenoid mononuclear cell infiltrates consistent with erythema multiforme (Figure 4). Patient was discharged home on November 3, 2007. The rash completely resolved within 9 days of eruption (Figure 5).

We performed a literature review searching for links between 1) EM, SJS, TEN and phenytoin and radiation therapy of all body systems except cranium and 2) EM, SJS, TEN and levetiracetam with radiation therapy of all body systems including cranium in PubMed and OVID MEDLINE databases. There is no article found in either link. However, there was one case report of SJS development at multiple sites of previously irradiated areas 2 weeks after lumbosacral radiation therapy while phenobarbital was administered concomitantly [10]. The patient received sequential localized radiation therapy at sacrum, cranium, thorax and lumbosacral areas at 6 months, 4 months, 1 month and 2 weeks prior to the eruption of SJS, respectively. He also received both phenytoin and phenobarbital about 3 months earlier for seizure treatment, but phenytoin was discontinued 42 days before the rash eruption.

The patient reported here had tolerated two previous radiation therapy treatments without development of skin toxicity. He also tolerated phenytoin treatment alone without any toxicity prior to starting the combined brain and spine radiation therapies. Since the eruptions occurred first in areas corresponding to the radiation treatment fields, it is likely that the SJS development is due to concurrent exposure to phenytoin and radiation treatments of brain and spine. The weaning of dexamethasone may contribute to the development of SJS as well. EM-SJS-TEN syndrome is well described in patients receiving concurrent phenytoin and cranial irradiation treatments, although there appears to be some confusion in the published literature whether it is erythema multiforme or SJS [11-13]. Ahmed and colleagues suggested an acronym "EMPACT" (Erythema Multiforme associated with Phenytoin And Cranial Radiation Therapy) to describe this reaction [11]. The case presented here demonstrates that the rash erupted in the patient's cranial radiation field and in the thoracic spinal irradiated areas, suggesting that phenytoin not only sensitizes for cranial radiation therapy, but also for radiation therapy received elsewhere in the body. It is possible that the combination of cranial radiation and phenytoin exposure triggered the sensitization and subsequent eruption of SJS on the scalp and on the skin of thoracic radiation fields. Combining the case reported by Duncan et al. [10], we believe that the EMPACT acronym may not cover the full spectrum of the syndrome because it suggests the syndrome is associated only with cranial irradiation with concurrent phenytoin administration.

It is believed that phenytoin induces cytochrome P450 3A and produces oxidative reactive intermediates that are involved in the hypersensitivity reaction [14]. Additionally, it is thought that the aromatic chain in the chemical structure of phenytoin and other agents undergo a detoxification pathway mediated by epoxide hydrolases [15]. Anticonvulsants that do not commonly cause SJS are metabolized differently. Since carbamazepine, valproic acid and barbiturates have shown cross-sensitivity with phenytoin, gabapentin was recommended as a substitute AED for a suspected sensitive patient while undergoing radiation treatment [16]. Gabapentin related SJS reactions, however, have been reported in the literature [16]. Nonetheless, they are rare events. Another alternative, levetiracetam, is increasingly being used as a phenytoin replacement [17]. Depending on the seizure types, there are many new AEDs with lower rate of rash formation used alone or with radiation which include valproic acid, topiramate and zonisamide [8,18,19]. Levetiracetam is not known to produce SJS, EM or TEN when administered alone or with concurrent radiation therapy. A search in PubMed and OVID MEDLINE for a link between the levetiracetam, radiation therapy and EM, SJS or TEN yielded no positive results. Furthermore, there is preliminary data that levetiracetam may be effective when used as a monotherapy of AED [20,21]. Therefore, for patients requiring AED who require radiation therapy, levetiracetam may be the preferred anticonvulsant, if there is no other contra-indication.

## Conclusions

We have described a case of SJS erupted in the fields of cranial and thoracic irradiation while receiving concomitant phenytoin therapy. This case demonstrates that SJS can occur in other areas of the body in addition to the cranium where radiation was delivered. For patients who need whole brain radiation and require anticonvulsant, phenytoin should be avoided. We suggest levetiracetam as a better substitute.

## Abbreviations

AED: Anti-Epileptic Drugs; SJS: Stevens-Johnson Syndrome; EMPACT: Erythema Multiforme associated with Phenytoin And Cranial Radiation Therapy; EM: Erythema Multiforme; TEN: Toxic Epidermal Necrolysis.

## Competing interests

The authors declare that they have no competing interests.

## Authors' contributions

AK and TD were involved in the study design, data analysis, writing and revision of the manuscript. JW and JM participated in clinical care and follow-up of the case patient, manuscript revision, and review of the intellectual content. JZ conceived of the study, participated in the study design, data analysis, manuscript revision and review of intellectual content. All authors read and approved the final manuscript.

## Consent

Written informed consent was obtained from the patient for publication of this case report and any accompanying images. A copy of the written consent is available for review by the Editor-in-Chief of this journal.
